# Different or the Same? Determination of Discriminatory Power Threshold and Category Formation for Vague Linguistic Frequency Expressions

**DOI:** 10.3389/fpsyg.2019.01559

**Published:** 2019-07-03

**Authors:** Franziska Bocklisch

**Affiliations:** Cognitive and Engineering Psychology, Department of Psychology, Technische Universität Chemnitz, Chemnitz, Germany

**Keywords:** discriminatory power, vague linguistic terms, frequency expressions, verbal uncertainty expressions, verbal response scales, membership functions, fuzzy pattern classification

## Abstract

In psychological research, many questionnaires use verbal response scales with vague linguistic terms (e.g., frequency expressions). The words’ meanings can be formalized and evaluated using fuzzy membership functions (MFs), which allow constructing distinct and equidistant response scales. The discriminatory power value of MFs indicates how distinct the functions and, hence, the verbal expressions are. The present manuscript interrogates the threshold of discriminatory power necessary to indicate a sufficient difference in meaning. Using an empirical validation procedure, participants (*N* = 133) estimated (1) three correspondence values for verbal expressions to determine MFs, and (2) similarities of words by pairwise comparison ratings. Results show a non-linear relationship between discriminatory power and similarity, and fuzzy MFs, as well as the searched-for threshold value for discriminatory power. Implications for the selection of verbal expressions and the construction of verbal categories in questionnaire response scales are discussed.

## Introduction

**FIGURE 1:**
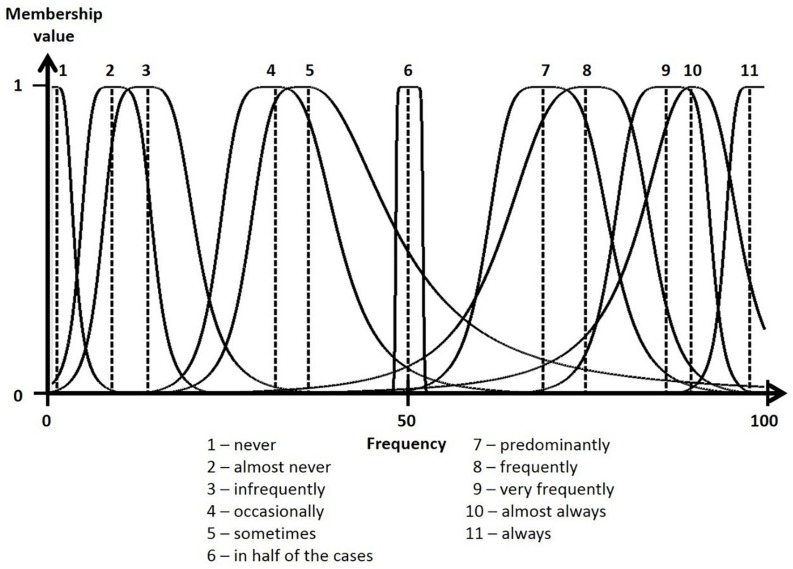
Membership functions of the 11 verbal frequency expressions.

The task of formalizing words’ meanings, such as in verbal expressions of frequency, intensity or probability, is relevant in a wide variety of research and application fields [e.g., verbal response categories and rating scales, risk and intercultural communication, medicine, forecasting, neuropsychological representation of words and numbers; cf. Teigen et al. (2013) for a review as well as Beyth-Marom (1982), Wallsten et al. (1986), Teigen and Brun (2003), Dhami and Wallsten (2005), and González et al. (2019)]. Hence, empirical estimation data, for example, numbers assigned to typical, minimum and maximum correspondence values for linguistic terms (LTs; cf. Bocklisch, 2011) are modeled using fuzzy membership functions (MFs; e.g., Zadeh, 1965; Budescu et al., 2003; Bocklisch et al., 2012) to preserve the inherent vagueness of LTs’ meanings. This vagueness precludes the determination of natural-language words precisely, for instance, in assigning a single correspondence number. As such, the notion of an interval of numbers that belong to an LT with varying memberships (see MFs in Figure 1) is considered more appropriate for description. The functional and beneficial nature of vagueness in many social situations (e.g., Erev et al., 1991) necessitates its inclusion as opposed to elimination. For example, Du et al. (2011) found that investors favor forecasts that are as precise as warranted by the information available, but not more precise.

The present paper utilizes MFs to describe LTs’ vagueness, and attempts to determine the discriminatory power (*dp*) threshold needed to indicate a sufficient difference in meaning. The *dp* of MFs describes how distinct functions are and is calculated based on the approximated overlapping area of the MFs (cf. Supplementary Figure S2). Based on theoretical considerations, Bocklisch et al. (2012) proposed a threshold of *dp ≥* 0.70. See pp. 148–149 in Bocklisch et al. (2012) for details concerning the approximation procedure and equations employed. Here, the *dp* threshold is validated empirically by a comparison to pairwise similarity ratings between LTs. In turn, implications for LT category formations are derived.

## Methods

### Participants

A total of 146 participants, mainly students from the Technische Universität Chemnitz (70% female, *M*_*Age*_*=* 27.5 years, SD_*Age*_*=* 10.9 years), took part in the study in return for course credit. Thirteen persons were excluded from the analysis because they did not understand the task. Additionally, in the course of outlier corrections, less than 2% of the estimation data were eliminated. The study was performed in accordance with relevant institutional and national guidelines and regulations (Chemnitz University of Technology, 2002; Deutsche Gesellschaft für Psychologie, 2018). Ethics committee approval was not required according to institutional guidelines, and informed consent was obtained from all participants. Participant anonymity and confidentiality of data use were ensured according to the EU’s General Data Protection Regulation.

### Materials and Procedure

The survey instrument was an online questionnaire consisting of three parts. In the first part, the numerical translation of LTs was implemented according to procedures proposed by Bocklisch et al. (2012). Briefly, participants were asked to assign three numerical values to each of the 11 frequency expressions (see translations from the original German in Table 1 and Figure 1). Then, they estimated (1) the typical value that best represented the given LT as well as (2) minimal, and (3) maximal correspondence values (e.g., the typical correspondence value for *frequently* is in ___ of 100 cases). These data were used to model fuzzy MFs (see Figure 1) and specify *dp* values (see Table 1). In short, I employed a parametric potential MF concept (one-dimensional, asymmetric version) with eight parameters, defined by the estimated correspondence data. A detailed description of MF type, theoretical background and justification, as well as equations, descriptions of parameters and parameter estimation algorithms, comparisons to other MF types (e.g., triangular MFs) and alternative modeling approaches can be found in Bocklisch and Hausmann (2018, pp. 300–304), Bocklisch et al. (2012, pp. 147–148), and Bocklisch et al. (2017, pp. 149–150).

**TABLE 1:** Discriminatory power values of membership functions (MFs) of frequency expressions.

**Frequency expression**	**1**	**2**	**3**	**4**	**5**	**6**	**7**	**8**	**9**	**10**	**11**
1. Never	0										
2. Almost never	0.78	0									
3. Infrequently	0.90	0.36	0								
4. Occasionally	0.99	0.97	0.88	0							
5. Sometimes	0.99	0.99	0.93	0.32	0						
6. In half of the cases	1.00	1.00	1.00	0.94	0.71	0					
7. Predominantly	1.00	1.00	1.00	0.96	0.82	0.98	0				
8. Frequently	0.99	0.99	0.97	0.92	0.77	0.93	0.20	0			
9. Very frequently	1.00	1.00	1.00	0.99	0.91	1.00	0.79	0.60	0		
10. Almost always	0.99	0.99	0.98	0.95	0.84	0.98	0.75	0.65	0.25	0	
11. Always	1.00	1.00	1.00	0.99	0.94	1.00	0.97	0.96	0.90	0.57	0

In the second part, subjects rated the similarity of the meaning of LT pairs (e.g., *sometimes* vs. *frequently*) on an 11-point scale (0 = completely different to 10 = completely identical). The third part consisted of demographic questions concerning age, sex, and main activity (cf. Spitzer, 2012).

## Results

### Fuzzy Analysis

Figure 1 shows the resulting fuzzy MFs for the LTs and their formalized meanings. Dotted lines mark the MFs’ positions and are equal to the MF parameter *r*, i.e., representative value (for a parametric description of the resulting MFs, see Supplementary Table S1) and are equal to the mean values of typical correspondence estimates. Mean minimal and maximal correspondence estimates were used to model the MFs’ expansions (i.e., *c_*l*_* and *c_*r*_* parameters characterizing left- and right-sided MF expansions). The remaining MF parameters (*b_*l*_, b_*r*_, d_*l*_, d_*r*_*), which determine a MF’s decline (shape) and hence its fuzziness, were modeled using the raw data (cf. Bocklisch and Hausmann, 2018). The resulting MFs are distributed along the numerical frequency scale in a non-equidistant manner. Furthermore, MFs’ shapes range from very narrow (e.g., LTs 1 and 6) to wide (e.g., LTs 5 and 8) indicating precise versus vague LT meanings. *Dp* values were calculated as a measure of difference in meaning. *Dp* values (see Table 1 and Supplementary Figure S1) were found to be high (*dp >* 0.70) for the majority of MFs except for the neighboring MFs *almost never*–*infrequently* (*dp =* 0.36), *occasionally*–*sometimes* (*dp =* 0.32), *predominantly*–*frequently* (*dp =* 0.20), *frequently*–*very frequently* (*dp =* 0.21), *frequently*–*almost always* (*dp =* 0.65), *very frequently*–*almost always* (*dp =* 0.25), and *almost always*–*always* (*dp =* 0.57). Hence, according to our theoretical definition (Bocklisch et al., 2012), these LTs are not sufficiently different in meaning.

### Discriminatory Power and Similarity

First, similarity data were recorded according to *dp* meaning so that small values indicate high similarity and high values indicate low similarity. The correlation between similarity and *dp* is rather high: *r =* 0.72. Supplementary Figure S1 shows the relationship between *dp* and similarity (raw data). The relation is non-linear and according to a closed mathematic function approach is best approximated by a cubical function. (*f*(*x*) = - 0.58 + 0.72*x^3^* - 0.11*x^2^* + 0.005*x*) A piecewise defined function (linear and/or non-linear) could describe the relation more flexibly. In order to specify the *dp* threshold value, the data point that combines medium similarity (5.13) and lowest *dp* (0.71) was identified (see Supplementary Figure S1, red dotted lines). This point was chosen due to practical implications (i.e., a threshold value that is not overestimated should indicate a difference in the semantic meaning of LTs). All similarity values >5.13 indicated differences in the LTs’ meanings and were related to *dp* values >0.71. Hence, the empirically sought-after threshold value for sufficiently distinct MFs/LTs meanings is *dp ≥* 0.71.

## Discussion

Results of the study are threefold:

(1) Previous empirical results for verbal frequency expressions could be replicated, with only small differences to the results reported by Bocklisch et al. (2012). The deviations in *r*-values (MFs positions) for LTs lie between 0.34 for *always* and 8.66 for *frequently* and the shapes are similar concerning precision and vagueness, respectively. Due to the large interindividual variability of estimates (e.g., Teigen and Brun, 2003), the group MF approach used here accounts for variability and potential contradictions in the estimation behavior of participants by using parameters (cf. Bocklisch et al., 2012). Our results indicate that the translation procedure and modeling of LTs’ meanings using MFs is applicable. Two remarkable differences related to *dp*s were observed: Bocklisch et al. (2012) found the LT pairs *almost never* and *infrequently* (*dp* = 0.86) as well as *almost always* and *always* (*dp* = 0.97) to be considerably different. This was not the case in the present study (see Table 1).

(2) The theoretically defined *dp* threshold of *dp ≥* 0.70 (Bocklisch et al., 2012) was empirically confirmed (*dp ≥* 0.71). Hence, LT pairs with *dp* values >0.70 are sufficiently semantically different. Both similarity measures, *dp* and similarity estimates, correlate (*r =* 0.72) but are based on different types of estimates. While similarity estimates are grounded on pair-wise comparisons of LTs, *dp*s are derived from direct estimates concerning single LTs. The relationship between *dp* and similarity estimates is thus not necessarily linear (cf. Supplementary Figure S1), because, among other reasons, pair-wise comparison estimates involve anchoring processes while direct estimations do not. Furthermore, the usage of the *dp* measure is reasonable for the selection of LTs and alternative difference measures, such as the distance between *r* values are not informative enough because they do not consider that the vague LTs overlap in meaning. For instance, MFs of *never* versus *almost never* and *almost never* versus *infrequently* show small differences in *r* distances (7.7 and 5.0) but large *dp* distinctions (0.78 vs. 0.36, see Supplementary Figure S2). Hence, *dp* represents a useful measure for the selection of LTs (e.g., for verbal response scales) and for LT category formation.
(3) As reported, some LTs show high similarity in meaning. Consequently, a scale of verbal frequency expressions need not comprise all 11 LTs but should rather include distinct LTs (cf. Bocklisch et al., 2012) and merged LTs that are non-distinct, within LT categories. Non-distinct LTs (see section “Fuzzy Analysis” and Table 1) were merged based on *dp* by averaging MFs’ parameters (see Supplementary Table S1). The resulting 7-point scale is composed of three single LTs and four LT categories (see Supplementary Figure S3). These LTs/LT categories are all sufficiently different. The categories are verbally labeled with the LTs they comprise (e.g., “almost never to infrequently”). While no more than the seven LTs/LT categories can be distinguished, scales with fewer LT grades are possible. Our results confirm findings of Kutscher et al. (2017), who suggest using a rating scale with a maximum of 6-points to better map categories on the measured latent trade (job satisfaction) compared to an 11-point scale. More experiments are needed that systematically address the questions of appropriate number of response categories, suitable verbal anchors and potentially influencing variables (e.g., context, cf. Pepper and Prytulak, 1974).

Findings of these studies are of potential interest for many fields of research and their application. Context is known to influence LTs’ meanings (Moxey and Sanford, 1993; Budescu et al., 2003). In order to account for context-related factors, such as base rates or values of events within the presented MF approach, the multidimensional version of the MF could be used (cf. Bocklisch and Hausmann, 2018).

An additional open question is whether an alternative modeling method to fuzzy MFs, such as probability density functions (PDFs), may be used. Meder and Mayrhofer (2017) model frequency LTs with PDFs as input for probabilistic models (e.g., in diagnostic reasoning) because MFs cannot be neatly integrated with probabilistic models of cognition. However, this claim is only partly accurate because the transformation of MFs into PDFs is indeed possible through normalization and parameter determination using search algorithms. Thereafter, Bayes modeling can follow, offering the advantage that all vagueness information contained in the original estimation data is preserved [not only mean and variance of typical correspondence values as shown by Meder and Mayrhofer (2017)]. Hence, a direct comparison between PDFs and MFs and an evaluation of the advantages and disadvantages based on results of Meder and Mayrhofer is not possible here, and should be pursued in future research.

## Ethics Statement

Ethical standards were followed in conducting the study. The study was carried out at Technische Universität Chemnitz in 2012 with students. The participants were fully informed about the study content prior to participation. The study was conducted as anonymous online questionnaire and all participants could withdraw from the study at any time.

## Author Contributions

FB conducted the experiment, analyzed the results, and wrote the manuscript.

## Conflict of Interest Statement

The author declares that the research was conducted in the absence of any commercial or financial relationships that could be construed as a potential conflict of interest.
