# Supplementary material for: Different or the Same? Determination of Discriminatory Power Threshold and Category Formation for Vague Linguistic Frequency Expressions
Source: Front Psychol. 2019 Jul 3;10:1559. doi: 10.3389/fpsyg.2019.01559 (PMC6616075; doi:10.3389/fpsyg.2019.01559)

Different or the Same? Determination of Discriminatory Power Threshold and Category Formation for Vague Linguistic Frequency Expressions

Franziska Bocklisch

Cognitive and Engineering Psychology, Department of Psychology, Technische Universität Chemnitz, Chemnitz, Germany

*** Correspondence:**Franziska Bocklisch
franziska.bocklisch@psychologie.tu-chemnitz.de

**Supplementary Material**

Table 1: *Parameters of Membership Functions (MFs) of Frequency Expressions*

| Frequency Expression | *r* | *b_l_* | *b_r_* | *c_l_* | *c_r_* | *d_l_* | *d_r_* |
| --- | --- | --- | --- | --- | --- | --- | --- |
| 1 Never | 0.70 | 0.00 | 0.69 | 0.65 | 1.91 | 20.00 | 3.35 |
| 2 Almost never | 8.40 | 0.56 | 0.56 | 4.31 | 5.56 | 5.12 | 5.06 |
| 3 Infrequently | 13.43 | 0.56 | 0.69 | 6.26 | 5.58 | 5.80 | 3.64 |
| 4 Occasionally | 31.32 | 0.56 | 0.75 | 7.70 | 6.45 | 5.44 | 3.21 |
| 5 Sometimes | 35.92 | 0.56 | 0.75 | 8.02 | 7.98 | 5.10 | 2.17 |
| 6 In half of the cases | 50.00 | 0.01 | 0.01 | 2.35 | 2.56 | 20.00 | 20.00 |
| 7 Predominantly | 68.87 | 0.62 | 0.62 | 7.26 | 8.58 | 4.66 | 4.33 |
| 8 Frequently | 74.77 | 0.69 | 0.56 | 9.32 | 9.06 | 3.06 | 5.06 |
| 9 Very frequently | 86.11 | 0.62 | 0.50 | 6.84 | 6.36 | 4.62 | 6.89 |
| 10 Almost always | 89.57 | 0.75 | 0.69 | 5.01 | 5.60 | 2.31 | 3.38 |
| 11 Always | 97.80 | 0.62 | 0.42 | 3.18 | 2.11 | 4.31 | 6.93 |

*Figure 1:* Relationship Between Discriminatory Power (*dp*) and Similarity Estimates in Data Distribution


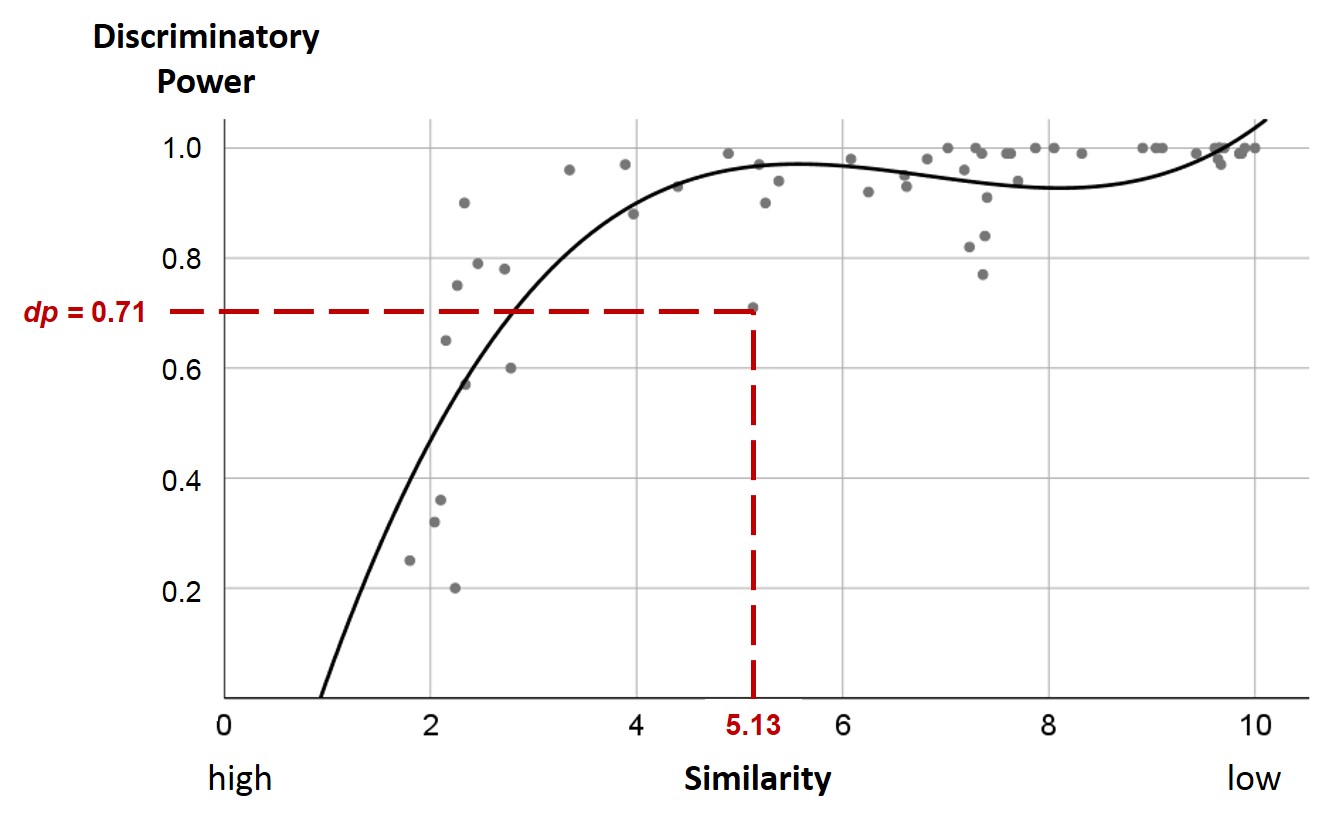


*Figure 2:* Relationship Between Discriminatory Power (*dp*) and Distance of Membership Functions


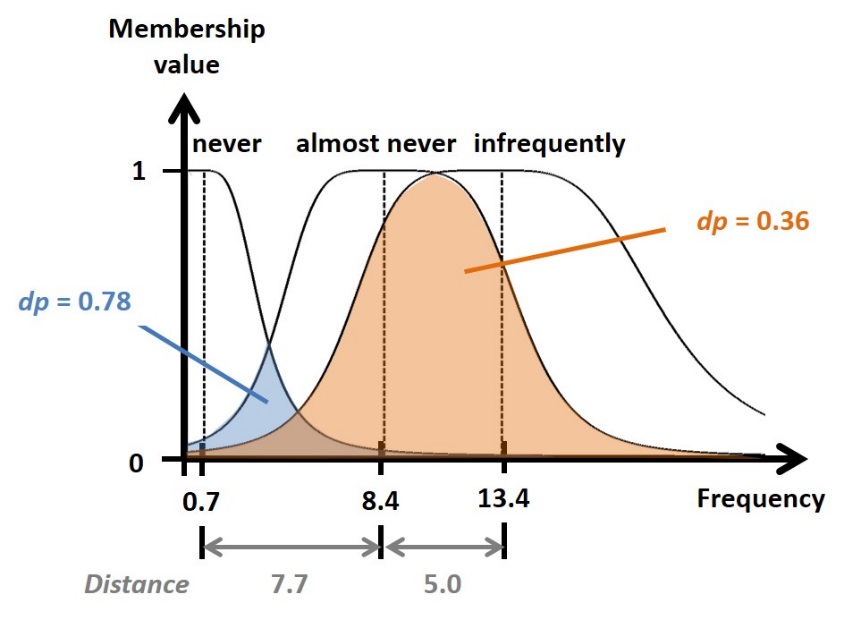


*Figure 3:* Membership Functions of Categories of Verbal Frequency Expressions and Discriminatory Power (*dp*)


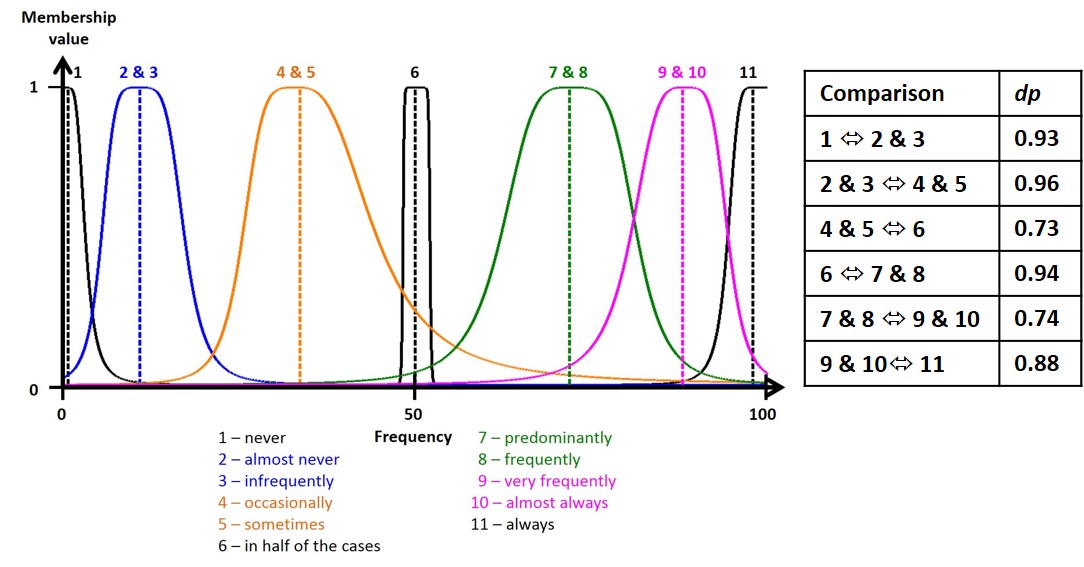

Supplement: Supplementary file 1 [file Data_Sheet_1.docx]
